# The shape of disposable diaper affects spontaneous movements of lower limbs in young infants

**DOI:** 10.1038/s41598-019-52471-4

**Published:** 2019-11-07

**Authors:** Hirotaka Gima, Midori Teshima, Etsuko Tagami, Toshihiro Sato, Hidenobu Ohta

**Affiliations:** 1 Child Developmental and Learning Research Center, Faculty of Regional Sciences, Tottori University, 4-101 Koyama-Minami, Tottori, 680-8551 Japan; 2 Global Research & Development Division, Unicharm Corporation, 1531-7 Wadahama, Toyohama-cho, Kanonji, Kagawa, 769-1602 Japan; 3 Department of Pyschophysiology, National Institute of Mental Health, National Center of Neurology and Psychiatry, 4-1-1 Ogawa-higashi-cho, Kodaira, Tokyo, 187-8553 Japan; 4 Department of Psychiatry, Asai Hospital, 38-1 Togane, Chiba, 283-0062 Japan; 5 Department of Neuropsychiatry, Akita University Graduate School of Medicine, 1-1-1, Akita, 010-8543 Japan

**Keywords:** Preventive medicine, Paediatric research

## Abstract

This study examined the characteristics of young infants’ lower limb spontaneous movements based on differences in shape of diapers. Twenty-seven healthy infants (103 ± 16.3 days old) were enrolled in this study. We measured the spontaneous movements of their lower limbs in four conditions (Naked, wearing Normal type diapers, wearing Type A diapers, and wearing Type B diapers). The Normal diaper has a wider waist belt than the Type A diaper, and the Type B diaper has a narrower crotch area than the Type A diaper. We observed them in seven indices (the velocity of lower limb movements, the trajectory area of knee movement in the sagittal plane and the frontal plane, the distance between both knees and between side of abdomen and knee, and correlation of velocities between side of abdomen and knee and between left and right ankles). The results showed that the velocity of the lower limb movements in the Naked condition was higher than when wearing Normal diapers. The value for the trajectory area of knee movement in sagittal plane, which reflects the range of lower leg lifting movements and closeness of such movements to the trunk, for the Type B diaper condition was higher than that for the Normal diaper condition. This result indicates that the shape of the diaper affects the spontaneous movements of the lower limbs of young infants.

## Introduction

Human fetuses and infants generate a variety of spontaneous movements and behaviors in early infancy^1,2^. These have attracted researchers’ attention and have been examined over recent years, because sensory-motor experience based on spontaneous movements and behaviors (such as non-object-oriented exploratory behaviors infants perform with their bodies) is considered important for early perceptual-motor development^3–9^. Ulrich *et al*.^3^ and Smith *et al*.^6^ showed that the quantity of kicking movements in infancy was related to the age at which they began to walk. Furthermore, Smith *et al*.^8^ indicated that there was less exploratory behavior of legs in infants at risk of developmental delay compared to infants with typical development, and Rademacher *et al*.^5^ reported that infants with myelomeningocele showed reduced leg movement activity. Furthermore, in the field of developmental psychology, evaluation of the movement characteristics of the lower limbs is used as a method for evaluating the memory and learning of motor (movement) in infants^10–12^.

In early infancy, it is possible that the shape and bulk of diapers has a significant effect on the spontaneous movements of the lower limbs. Most infants wear diapers during the first few years of life. Cole *et al*.^13^ reported that diapers reduced the proficiency of infants’ gait in 13 and 19-month-olds (steps of a diaper-less (naked) group were straight and narrow, but steps of a diaper-wearing group were noticeably wider and less mature). Regarding early infancy, it is possible that the shape and bulk of diapers has a significant effect on the quantity and/or quality of infants’ spontaneous movements. Previous studies also suggested that a seasonality effect^14,15^ and influence of clothes and bedclothes^16^ affects infants’ gross motor development (i.e. roll, creep, crawl, foot grasping and playing with toys). These studies indicated that restriction of spontaneous movements and mobility reduce levels of physical activity, and fewer opportunities for physical activity may constrain the acquisition of motor development in early infancy.

The assessments of the quality of infants’ spontaneous movements based on visual assessment have provided insights into the functional integrity of infants’ central nervous system, leading to the delineation of developmental profiles that may be useful in the detection of neurological deficits in early infancy^17–20^. Currently, in addition to visual assessment, evaluation also uses computer-based video analysis^21–26^, 3-dimensional motion analyzers^27–31^, tri-axial accelerometers^32,33^, and magnetic tracking systems^34^. These studies are necessary for the understanding of the developmental process and mechanisms in infants’ motor development, and contribute to early detection and intervention in developmental disorders during early infancy. However, in previous studies, little attention has been given to the influence of the shape of diapers on spontaneous movements.

To our knowledge, analysis using digital devices on influence of the shape and bulk of disposable diapers on quantity and/or quality of spontaneous movements in young infants has not yet been reported in literature. In this study, we examined whether different shape characteristics of diapers affect spontaneous movements of the lower limbs of young infants of approximately 3 months of age. We hypothesized that the shape of either the waistband around the abdomen or the crotch area between the knees affects the spontaneous movements of the lower limbs of young infants. If the shape and bulk of the diaper affects the movement characteristics of the lower limbs, it is an important factor to be considered for the infant’s sensory-motor experience and later motor development. It is hoped that the results of our study will lead us to be able to determine better clothing designs for infants that do not hinder their spontaneous and natural body movements.

## Results

**Figure 1:**
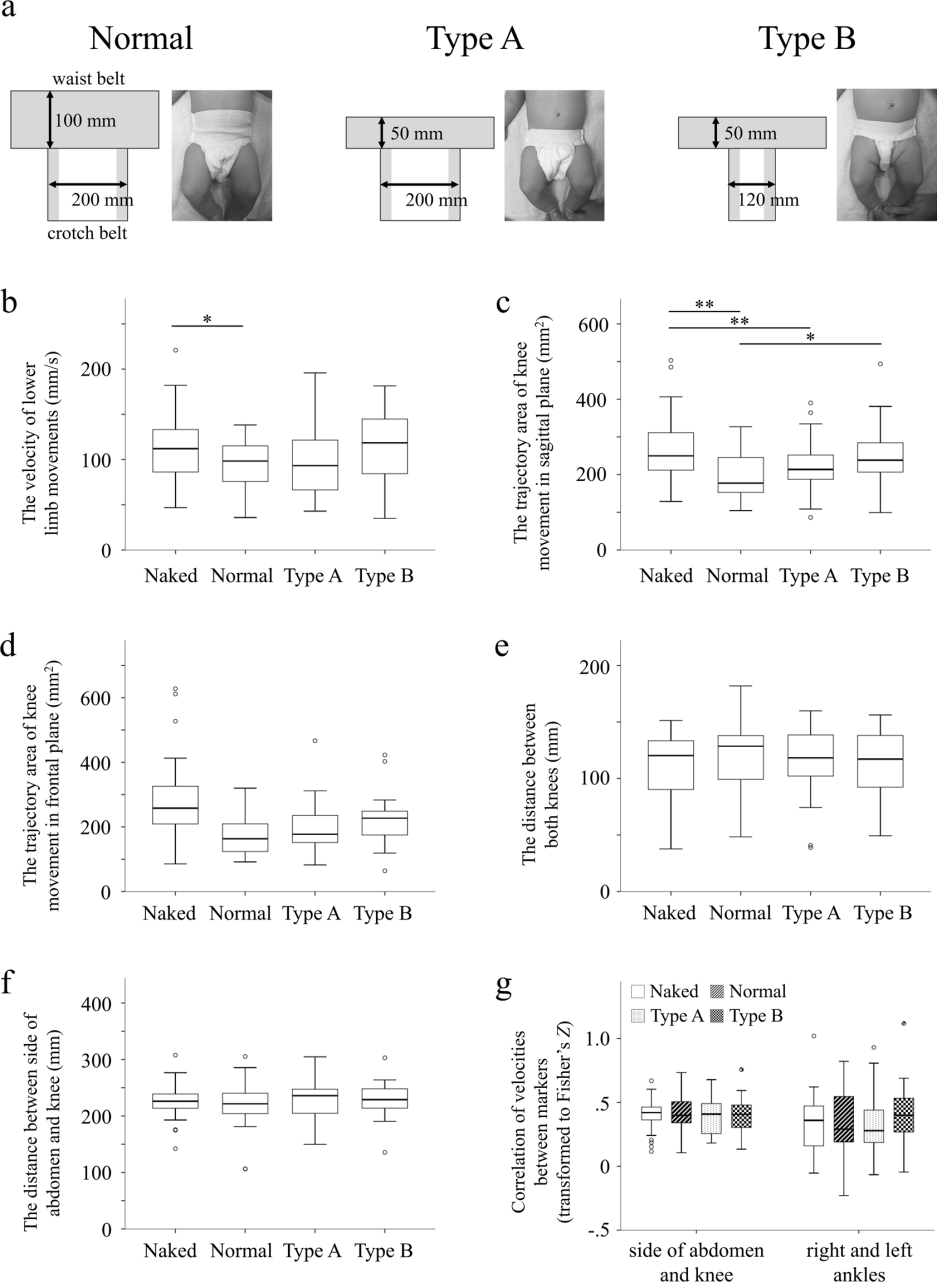
Specifications of each diaper and the values of the indices of the lower-limb movements in each dress-condition. (**a**) The dimensions of each diaper. Elastic material is used in the gray part of the illustration of each diaper. (**b**) The Velocity of lower-limb movements. (**c**) The trajectory area of knee movement in sagittal plane. (**d**) The trajectory area of knee movement in frontal plane. (**e)** The distance between both knees. (**f**) The distance between side of abdomen and knee. (**g**) Correlation of velocities between side of abdomen and knee, and between left and right ankles. Median, 25th and 75th percentiles and range are shown. *p < 0.05; **p < 0.01.

The size of each diaper and the median values of each index for each dress-condition are shown in Fig. 1. Figure 1a describes the designs of the three types of diapers (Normal, Type A and Type B) used in the present study. Figure 1b shows the average velocity of lower limb movement in each dress-condition. The Friedman test showed significant inter-group differences in average velocity of lower limb movement (p = 0.012). The post hoc comparison revealed that the average velocity of lower limb movement in the Naked condition was significantly higher than that in the Normal condition (p = 0.046). Figure 1c,d shows the trajectory area of knee movement for each dress-condition. The Friedman test showed significant inter-group differences in the trajectory area of knee movement in sagittal plane (p = 0.0001). The post hoc comparison revealed the trajectory area of knee movement in sagittal plane in the Naked condition was significantly higher than that in the Normal (p = 0.0008) and the Type A condition (p = 0.008). Furthermore, the value for the Type B condition was also significantly higher than that for the Normal condition (p = 0.032). There was no significant inter-group difference in the trajectory area of knee movement in the frontal plane. Figure 1e,f show the results of the average distance between both knees, and the average distance between the abdomen and knee, respectively. Note that, in calculating the average value of each index, we conducted a mixed 4 (dress-condition) × 2 (left and right legs as within-subject variables) ANOVA the velocity of lower limb movements and confirmed that there were no significant main effects of left and right legs. There was no significant intergroup difference in the Friedman test for values of the average distance between both knees, and the average distance between the abdomen and knee. Figure 1g show correlation of velocities between the side of the abdomen and knee and between left and right ankles in each dress-condition, there was no significant intergroup difference.

## Discussion

The present study is the first to demonstrate that we can allow infants to move their lower limbs more freely by making both the waist and crotch belts of diapers narrower than those presently used but not by just making the waist belt narrower.

The data indicate that wearing a diaper with a normal shape may restrict the lower-limb movements of young infants. Figure 1b,c demonstrate that the values of velocity of lower limb movements and the trajectory area of knee movement in sagittal plane of infants wearing Normal diapers were significantly lower than for infants in Naked condition, suggesting that infants in Naked condition moved their legs more freely compared to when wearing Normal diapers. Furthermore, the value of the trajectory area of knee movement in sagittal plane in Type A condition was significantly lower than that in Naked condition, suggesting that the crotch width of the diaper affects the range of the infant’s lower limb movements.

The present results also suggest that the diaper waistband width interacted with the diaper crotch width to affect the spontaneous movements of the lower limbs of young infants. Figure 1c demonstrates that the trajectory area of knee movement in sagittal plane of infants wearing Type B diapers was significantly greater than that of infants wearing Normal diapers. Normal diapers and Type B diapers differ in waistband width (Normal = 100 mm, Type B = 50 mm) and crotch width (Normal = 200 mm, Type B = 120 mm). In contrast, despite there being a difference in waistband width between Normal and Type A diapers, no significant difference in any of the parameters (the velocity of lower limb movements, the trajectory area of knee movement in the sagittal plane and the frontal plane, the distance between both knees and between side of abdomen and knee, and correlation of velocities between side of abdomen and knee and between left and right ankles) was detected between infants in Normal and Type A diapers (Fig. 1b–g), suggesting that waistband width alone does not affect the movement of the lower limbs of infants.

Consistent with the present study, previous studies have reported that the shape of diapers plays an important role in the development of spontaneous movements of the lower limbs (antigravity limb movements) of young infants. Cole *et al*.^13^ showed that walking is adversely affected by the wearing of disposable diapers. They reported that infants wearing diapers displayed less mature gait patterns and made more missteps and falls. Groenen *et al*.^35^ reported that not only diapers but also clothing can affect the frequency and quality of young infants’ movement. In these studies, the influence of diaper wearing and shape on the movement characteristics of infants was evaluated using data on walking and steps. It is difficult to directly compare the results of these studies with our research. However, previous studies showed that the quantity of kicking movements in infancy was related to the age at which infants began to walk^3,6^, and indicated that there was less exploratory behavior of legs in infants at risk of developmental delay compared to infants with typical development^8^. The difference between diaper type in the amount (the velocity) and range (the trajectory area of knee movements in sagittal plane) of lower limb movements in the supine position in early infancy shown in this study may possibly affect walking characteristics. The present study supports a possibility that more advanced design of infant clothing, including diapers, may contribute to more proper development of body movements of young infants.

It is important to note that the findings of the present study are limited. We have demonstrated a difference in one of the parameters of lower limb spontaneous movements between the Type B and Normal diaper condition, but we did not detect any difference in the other parameters of lower limb movements among the Type A, Type B, and Normal diaper conditions (Fig. 1b–g).

Our findings may be insufficient to conclude that the results of the present study will lead us to be able to determine better clothing designs for infants that do not hinder their spontaneous and natural body movements. However, even with its limited results, the present study indicates that diaper design has a significant effect at least on the trajectory area of knee movement in the sagittal plane - a probable indicator of the range of lower leg lifting movements and proximity of these movements to the trunk. The extent to which such movement reflects the amount of kicking in the lower limbs needs further study, but it has also been reported that the development of antigravity movements are critical for infants as they promote exploration activities of the form of their body, and increase sensory-motor experiences such as hand-foot contact (double touch), which are important as a basis for later motor development^9,28,30^. As diaper design can influence such movements, it seems possible that careful design of infant clothing, including diapers, may contribute to more proper development of the body movements of young infants. Adjustment of diaper shape could potentially be utilized for not only preterm infants, but also for term infants who happen to have insufficient antigravity limb movements in early infancy. Miyagishima *et al*.^28^ reported that antigravity limb movements in preterm infants within the first 3 months of corrected age were insufficient compared with those in term infants, and no improvement in development was observed in preterm infants within those first 3 months. They also showed that gross motor development at 6 and/or 12 months corrected age was delayed in infants who had insufficient antigravity limb movements in early infancy^30^.

We also acknowledge that this study may not include all possible parameters which might significantly reflect lower limb movements in infants. However, this study has demonstrated that the combined effects of narrower waist and crotch belts can affect infants’ lower limb movements in terms of trajectory area. In future research, it will be beneficial to identify other parameters such as other movements related to hip joint movements which may affect infants’ lower limb movements by utilizing more sophisticated three-dimensional data. In addition, this study does not evaluate how strongly the shape of diapers affects the postnatal development of the lower limb movement of older infants since we only assessed the movement at the one developmental stage of approximately 3 months of age and not later developmental stages. Also, because of the relatively small sample size (n = 27), this study did not fully perform analysis on sex difference in the parameters. Although the study population is also well-balanced between boys (n = 14) and girls (n = 13), a study population with a larger sample size will be required to make a satisfactory statistical analysis of the effect of sex difference on the parameters in a future study.

## Methods

### Participants

The characteristics of the participating infants are shown in Table 1. Twenty-seven healthy infants (14 boys and 13 girls, 103 ± 16.3 days old) participated in this study. Infants at birth were of 39.4 ± 1.1 weeks gestational age. There was no diagnosis of neurological disorder at the time of measurement in any of the participants.

**Table 1:** Characteristics of participating infants.

Male/female (n)	14/13
Weeks of gestational age (weeks), (mean ± SD)	39.4 ± 1.1
Birthweight (g), (mean ± SD)	3024.5 ± 399.3
Age at recording of spontaneous movements (days), (mean ± SD)	103 ± 16.3
Weight at recording of spontaneous movements (g), (mean ± SD)	6299.3 ± 1024.7

SD; standard deviation.

Ethical approval for this study was obtained from the ethical committee of the Japan Developmental Care Research Association (JDCRAE2016-01) and all procedures were performed in accordance with the approved guidelines and regulations. Written informed consent was obtained from the parents for data collection and any type of publication including online open-access publication. The parents agreed to their child’s participation in the study with full knowledge of the experimental nature of the research.

### Procedure

#### Recording of spontaneous movements

**Figure 2:**
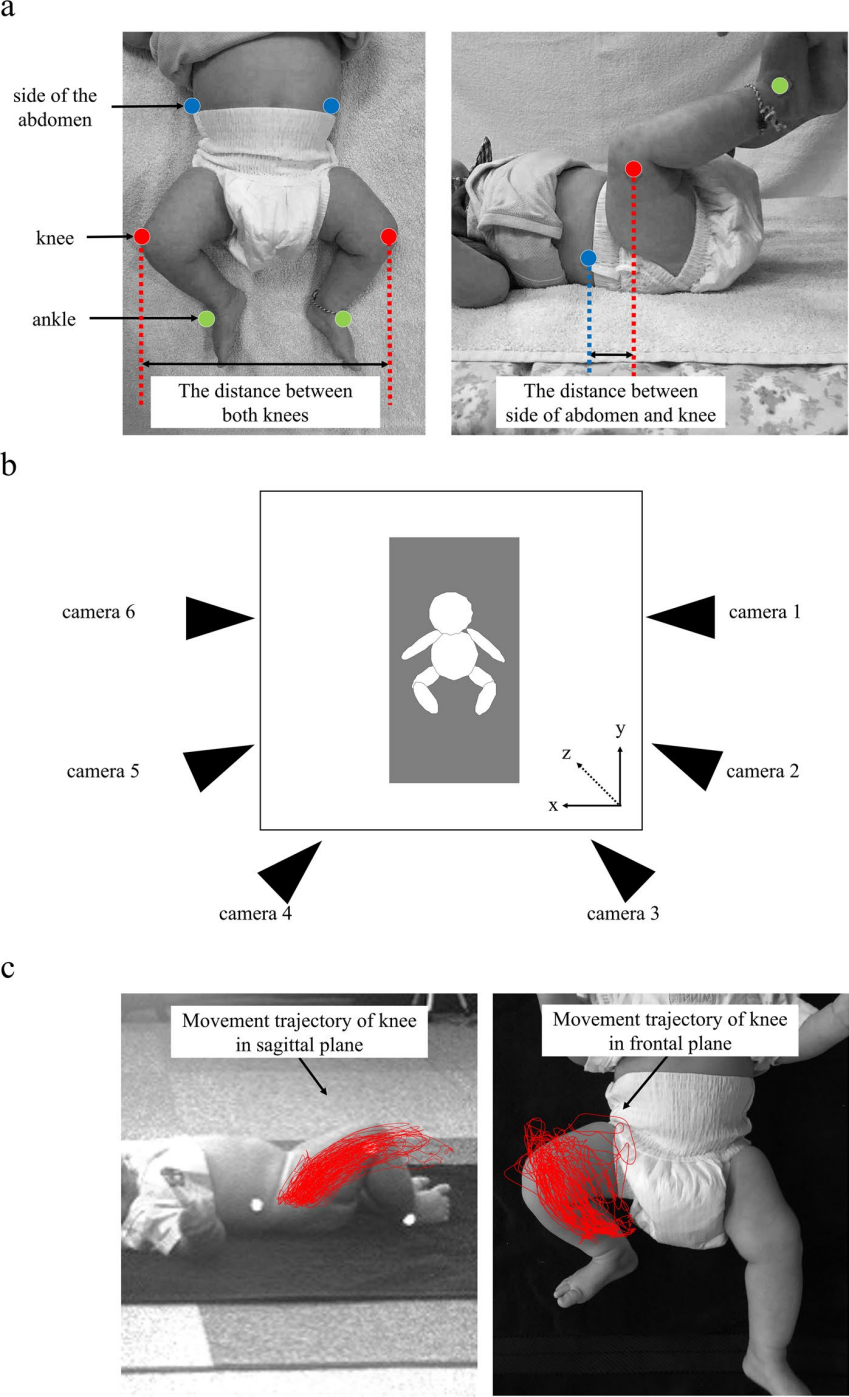
Motion analysis system and measurement setup, marker attachment position, movement trajectory of knee, and the distance between markers. (**a**) The marker attachment position and the distance between both knees and between side of abdomen and knee (right side). (**b**) Measurement environment and position of CCD monochrome-shuttered cameras. (**c**) Movement trajectory of knee in sagittal plane and frontal plane (right side).

The infants underwent observation in a laboratory at Unicharm Corporation. They were positioned on their backs on a baby mattress during the experiment, and hemispherical reflective markers (diameter, 15 mm; weight, 1 g) were attached to the following positions on both side of each infant; horizontal edge of horizontal line of the umbilicus (side of the abdomen), the head of fibula (knee), and the lateral malleolus (ankle) (Fig. 2a). We measured the spontaneous movements of their lower limbs in the following four conditions: wearing nothing (Naked), wearing a normal type diaper (Normal), wearing an A type diaper (Type A), and wearing a B type diaper (Type B). The measurements of each diaper are shown in Fig. 1a. The order in which the 4 measurement conditions were employed was determined randomly using a random number table, and diapers were changed by researchers. Spontaneous movements of the lower limbs of the infants in each condition were measured for 5 minutes while the infants were in an awake, active, and non-crying state. The infant’s arousal state for data collection was defined by the Neonatal Behavioral Assessment Scale^36^ and data collection was terminated whenever infants cried. There were no infants who fell asleep during measurement. All data was collected after two trials with each infant. The average duration for data collection for each infant was 29.6 ± 6.2 minutes. The infants could not see their parent(s) or the experimenters during the sessions.

From the measured 5 minutes measured for each condition, we selected a 2-minute period for analysis in which continuous movement occurred for analysis. The movements of the end points of the infants’ lower extremities were recorded in a three-dimensional (3D) space using a 3D motion capture system (Library, Tokyo, Japan). Six Charge-Coupled Device (CCD) monochrome-shuttered cameras (sampling frequency of 120 Hz) were placed around the mattress on which the infants were positioned (Fig. 2b). The video images captured by the cameras were digitized using video processors, and the 3D position of each marker was tracked. The marker-coordinated time-series data were smoothed using a 6-Hz recursive Butterworth filter algorithm.

### Data analysis

We used the time series data of the 3D positions (x, y, and z) of each marker (sampling frequency: 60 Hz), and examined the following indices^11,27,28^:

#### Velocity of lower limb movements

The movement distance for each frame in an experimental period was calculated from the 3D ankle marker position data. The distance was then divided by time (for every 1/120 sec) to calculate the time series velocity of each frame. Then, we averaged the time series velocities of all of the frames to calculate the average velocity of lower limb movements of each infant. The values were calculated separately for each side, and bilaterally averaged values were calculated. This value reflects the amount of movements in the lower limbs.

#### The trajectory area of knee movement

We traced the movements of the left and right knee markers using the analysis tool of the 3D motion capture system and saved the trajectory viewed from the sagittal plane and frontal plane as an image (Fig. 2c). Then, using an image analyzer (ImageJ, NIH, http://imagej.nih.gov/ij/), the trajectory area for the left and right knee was calculated, and this was averaged to determine the value of the trajectory area of knee movement. The value was calculated separately for the sagittal and frontal planes.

#### The distance between both knees, and between the abdomen and knee

The distances between the right and left knee markers, and between side-of-abdomen markers and knee markers were measured (Fig. 2a). We used only active periods (when the velocity of the ankle marker exceeded 30 mm/s) within a selected 2-minute period from the entire time series of marker-position data to calculate each index. For the value of the distance between both knees, the average value in the active period was calculated. For the value of the distance between side of abdomen and knee, the average values in active period was calculated separately for each side, and bilaterally averaged values were also calculated. In calculating the average value of each index, we conducted a mixed 4 × 2 ANOVA (Naked, Normal, Type A, and Type B × left and right legs as within-subject variables) the velocity of lower limb movements in order to confirm the bilateral difference for spontaneous movement of legs. There were no significant main effects of left and right legs (F = 1.67, p = 0.208), but a significant main effect of dress-condition (F = 3.211, p = 0.028).

#### Correlation of velocities between side of abdomen and knee, and between left and right ankles

To examine the coordination among the movements of each part, we calculated the Spearman’s rank correlation coefficient between the various velocity data of the entire time series. This index measures the degree of similarity in the waveforms of the velocities produced by each marker. We calculated the correlation between the velocity data for the side of abdomen and the knee. The values were calculated separately for each side, and bilaterally averaged values were calculated. Further, we calculated the correlation between the velocity data of the left and right ankles.

### Statistical analysis

We used the Statistical Package for the Social Sciences software, version 23 (SPSS IBM Japan Inc., Tokyo, Japan) for statistical analysis. We used Friedman’s test for comparisons between the four dress-conditions (Naked, Normal, Type A, Type B) for each index, because some indices did not show normal distribution based on the Shapiro-Wilk test (Table 2). When Friedman’s test showed significant results, the Bonferroni post hoc test was used as a multiple comparison. Regarding the correlation of velocities, we used Fisher’s *Z* score for comparing the correlation coefficients in each dress-condition.

**Table 2:** Test of normality of indices by Shapiro-Wilk test.

	Naked		Normal		Type A		Type B	
	*W*	*p*	*W*	*p*	*W*	*p*	*W*	*p*
The velocity of lower limb movements	0.97	0.51	0.97	0.60	0.94	0.10	0.98	0.73
The trajectory area of knee movement in sagittal plane	0.93	0.08	0.95	0.23	0.94	0.14	0.95	0.22
The trajectory area of knee movement in frontal plane	0.88	0.01	0.94	0.10	0.85	0.00	0.91	0.02
The distance between both knees	0.96	0.30	0.90	0.02	0.98	0.86	0.94	0.13
The distance between side of abdomen and knee	0.94	0.12	0.97	0.49	0.91	0.02	0.94	0.10
Correlation of velocities between side of abdomen and knee	0.95	0.23	0.98	0.81	0.94	0.13	0.98	0.77
Correlation of velocities between left and right ankles.	0.94	0.12	0.94	0.13	0.93	0.05	0.94	0.14
